# Supplementary material for: Extensive DNA methylome rearrangement during early lamprey embryogenesis
Source: Nat Commun. 2024 Mar 4;15:1977. doi: 10.1038/s41467-024-46085-2 (PMC10912607; doi:10.1038/s41467-024-46085-2)
Supplement: Supplementary file 1 — Supplementary Information [file 41467_2024_46085_MOESM1_ESM.pdf]

# Extensive DNA methylome rearrangement during early lamprey embryogenesis

Allegra Angeloni[1,2], Skye Fissette[3], Deniz Kaya[4], Jillian M. Hammond[5,6], Hasindu Gamaarachchi[5,6,7], Ira W. Deveson[5,6,8], Robert J. Klose[4], Weiming Li[3], Xiaotian Zhang[9,10], Ozren Bogdanovic[1,2,11]

[1] Garvan Institute of Medical Research, Sydney, Australia
[2] School of Biotechnology and Biomolecular Sciences, University of New South Wales, Sydney, Australia
[3] Department of Fisheries and Wildlife, Michigan State University, East Lansing, United States
[4] Department of Biochemistry, University of Oxford, Oxford, United Kingdom
[5] Genomics Pillar, Garvan Institute of Medical Research, Sydney, NSW, Australia.
[6] Centre for Population Genomics, Garvan Institute of Medical Research and Murdoch Children's Research Institute, Australia.
[7] School of Computer Science and Engineering, University of New South Wales, Sydney, NSW, Australia.
[8] Faculty of Medicine, University of New South Wales, Sydney, NSW, Australia.
[9] Center for Epigenetics, Van Andel Research Institute, Grand Rapids, United States
[10] Current address: University of Texas Health Science Center, Houston, TX, United States
[11] Centro Andaluz de Biología del Desarrollo, CSIC-Universidad Pablo de Olavide-Junta de Andalucía Seville, Spain

## Supplementary Figures S1-S5
## Supplementary Tables S1-S3

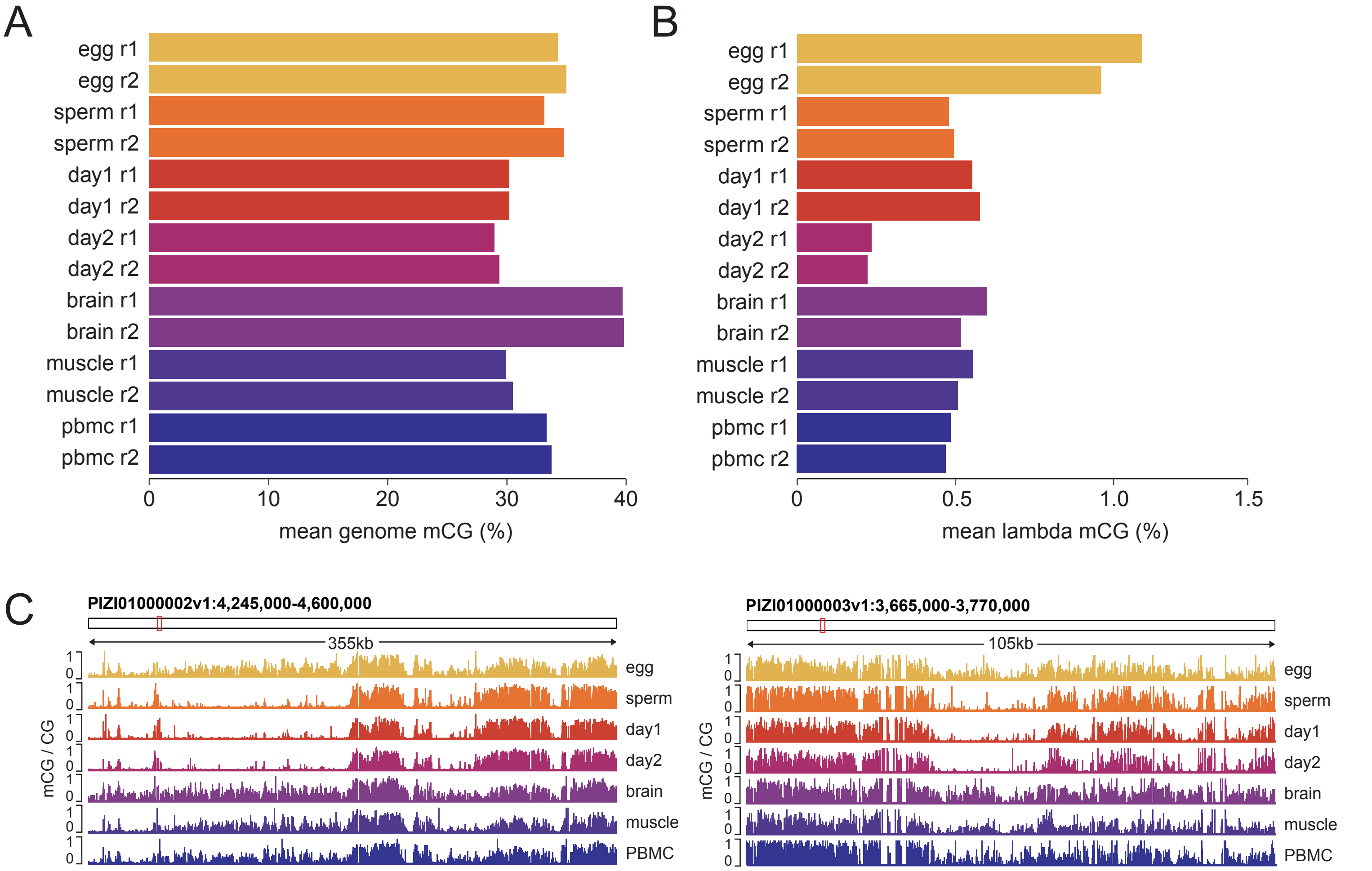


**Supplementary Figure S1. Global DNA methylation levels in seven lamprey tissues.** r1 = WGBS replicate 1, r2 = WGBS replicate 2. **A)** Mean genomic mCG levels. **B)** mCG levels in the unmethylated lambda phage DNA spike-in control for every WGBS dataset generated for this study. **C)** Browser tracks depicting mCG profiles. mCG values are from merged WGBS replicates.

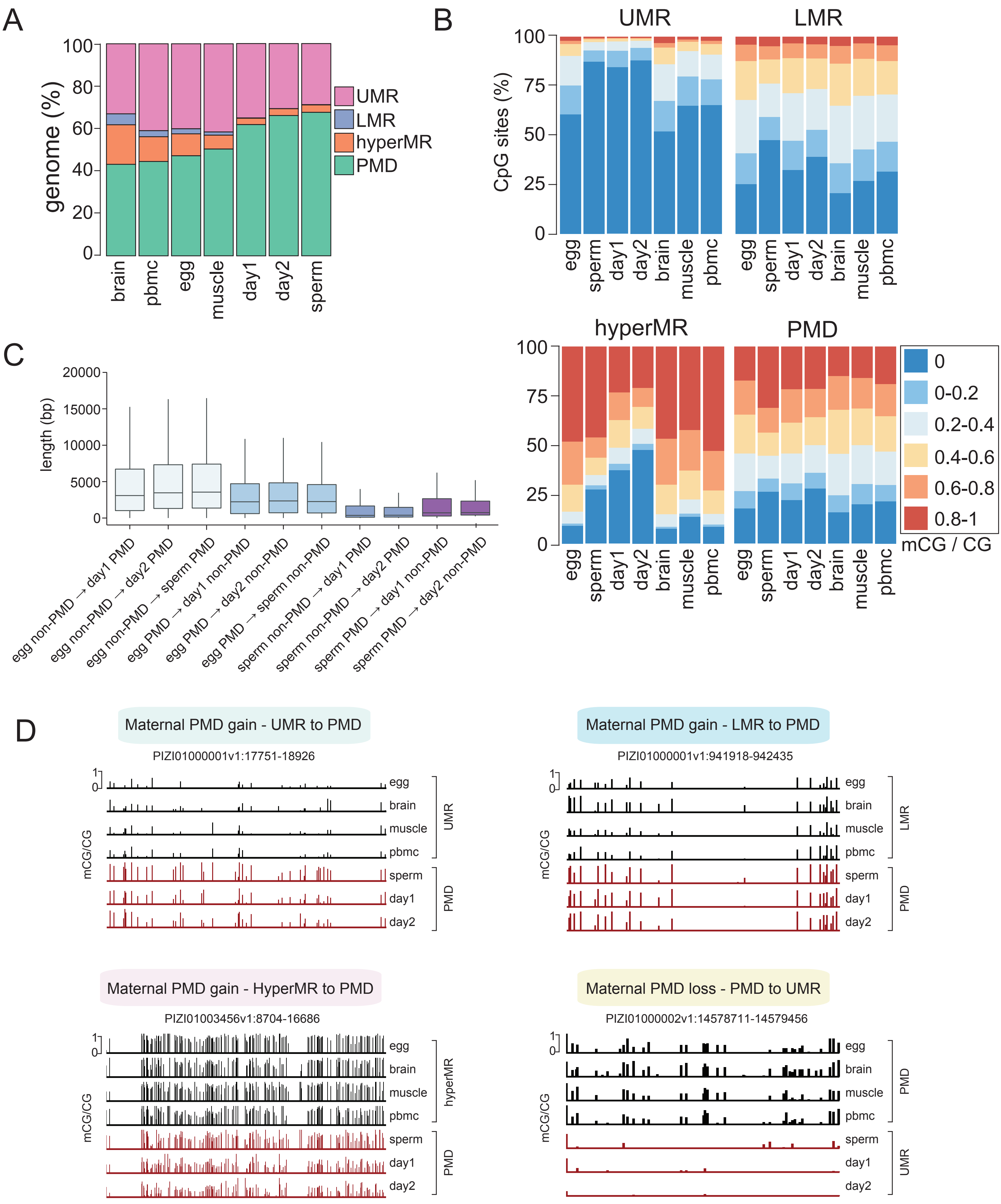


**Supplementary Figure S2. Sequence features of lamprey PMDs. A)** Percentage of the lamprey genome covered by PMDs and non-PMDs (unmethylated regions (UMRs), low methylated regions (LMRs), hypermethylated regions (hyperMRs). **B)** Percentage of CpG sites displaying low (0, 0-0.2), intermediate (0.2 - 0.4, 0.4 - 0.6, 0.6 - 0.8) and high (0.8 - 1.0) mCG levels at PMDs and non-PMDs in embryonic and adult somatic and germline tissues. mCG levels in this figure are from merged WGBS replicates. **C)** Distribution of sequence lengths of developmentally reprogrammed PMDs. The boxes show the interquartile range (IQR) around the median. The upper and lower whiskers extend from the hinge to the largest and smallest value, respectively, no further than 1.5 IQR. **D)** Browser tracks depicting mCG profiles of reprogrammed PMDs.

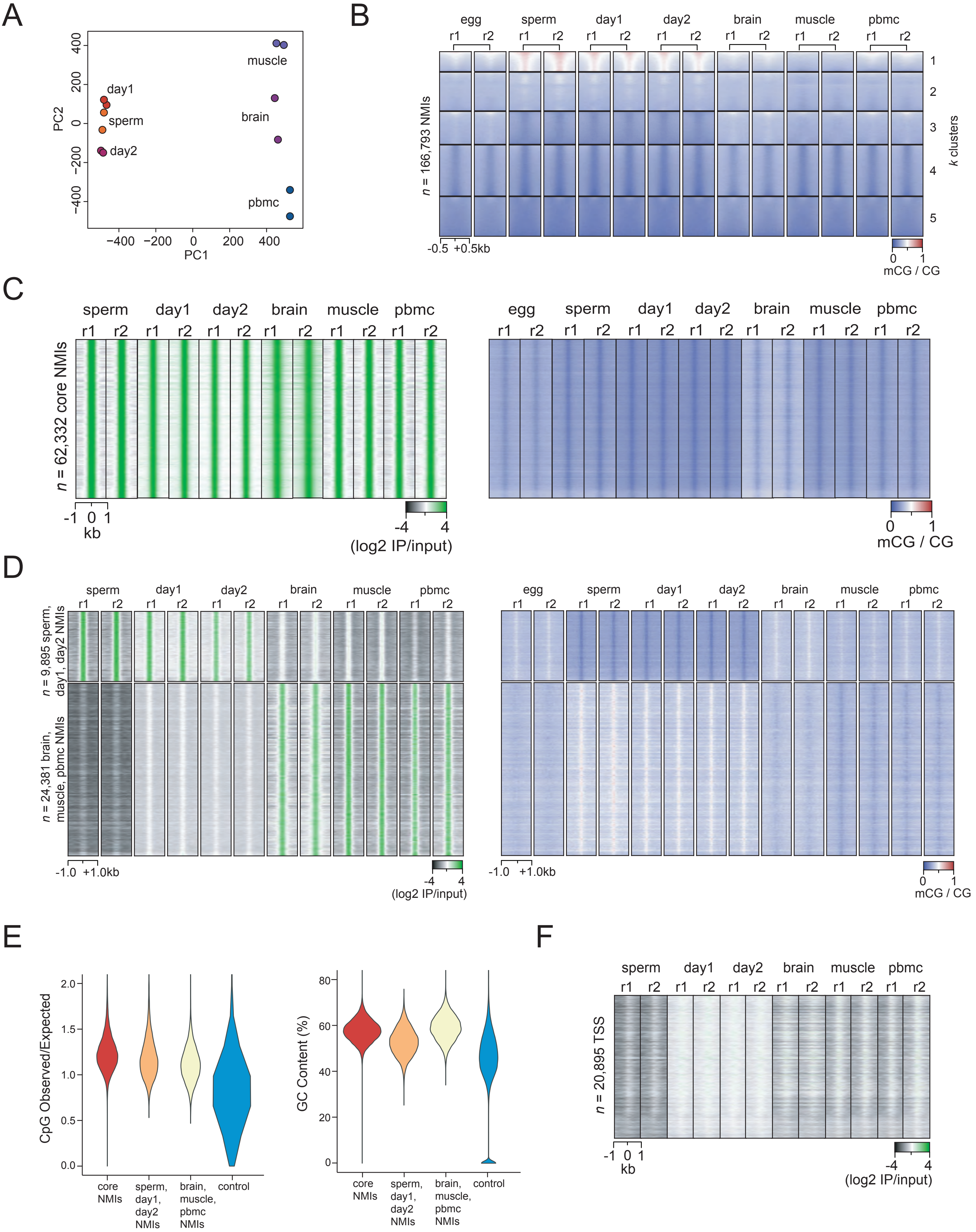


**Supplementary Figure S3. Sequence and epigenetic features of shared and tissue-specific NMIs. A)** PCA of normalized BioCAP read density (in replicate) at NMIs. **B)** *K*-means clustered mCG signal at merged NMIs ($k$ = 5). Y-axis boxes refer to individual clusters. r1 = replicate 1, r2 = replicate 2. **C)** BioCAP and mCG signal at core NMIs. **D)** BioCAP and mCG signal at NMIs enriched in sperm, day 1 and day 2, and at NMIs enriched in brain, muscle and PBMC. **E)** Distribution of CpG observed/expected ratio and GC content at: core NMIs; NMIs enriched in sperm, day 1 and day 2; NMIs enriched in brain, muscle and PBMC; and random control sequences. **F)** BioCAP signal at transcription start sites of protein-coding genes.

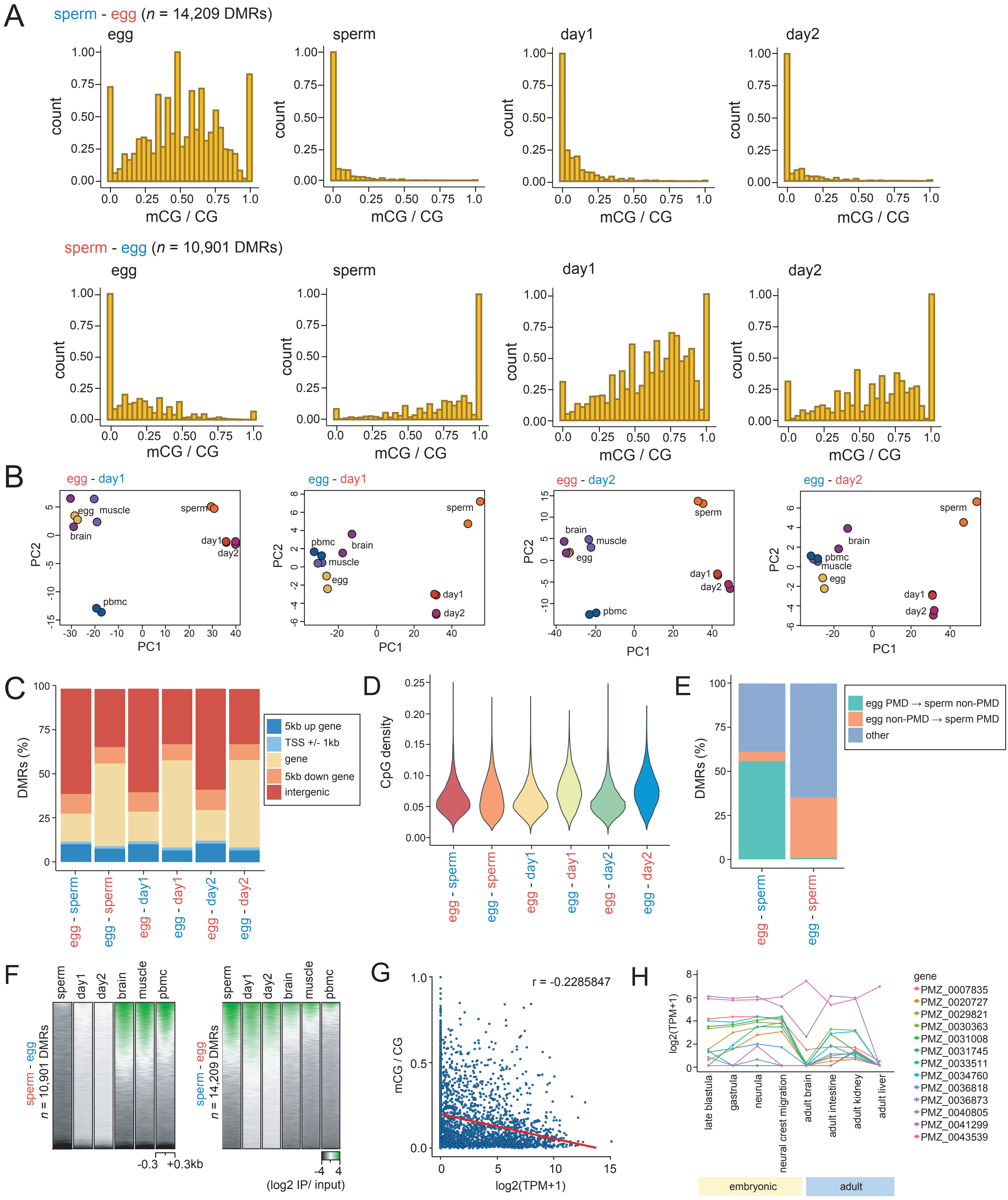

**Supplementary Figure S4. Maternal to paternal reprogramming at differentially methylated regions (DMRs) during lamprey embryogenesis. A)** Distribution of mCG levels at egg/sperm DMRs (blue = hypomethylation; red = hypermethylation). mCG values are from merged WGBS replicates. **B)** PCA of mCG levels from WGBS data (in biological replicate) at egg/day 1 and egg/day 2 DMRs. **C)** Percentage of egg/sperm, egg/day 1 and egg/day 2 DMRs overlapping diverse genomic features. **D)** Distribution of CpG density at egg/sperm, egg/day 1 and egg/day 2 DMRs. **E)** Percentage of egg/sperm DMRs overlapping egg/sperm PMDs. **F)** BioCAP signal at egg/sperm DMRs. BioCAP signal is depicted in descending order of signal intensity. **G)** Mean mCG levels (from merged brain WGBS data) of protein-coding gene promoters containing an NMI directly overlapping the TSS (*n* = 5188 genes) compared to expression levels of the associated gene in adult brain tissue. r = correlation coefficient. **H)** Expression patterns of genes with differentially methylated promoters in embryonic and adult tissues.

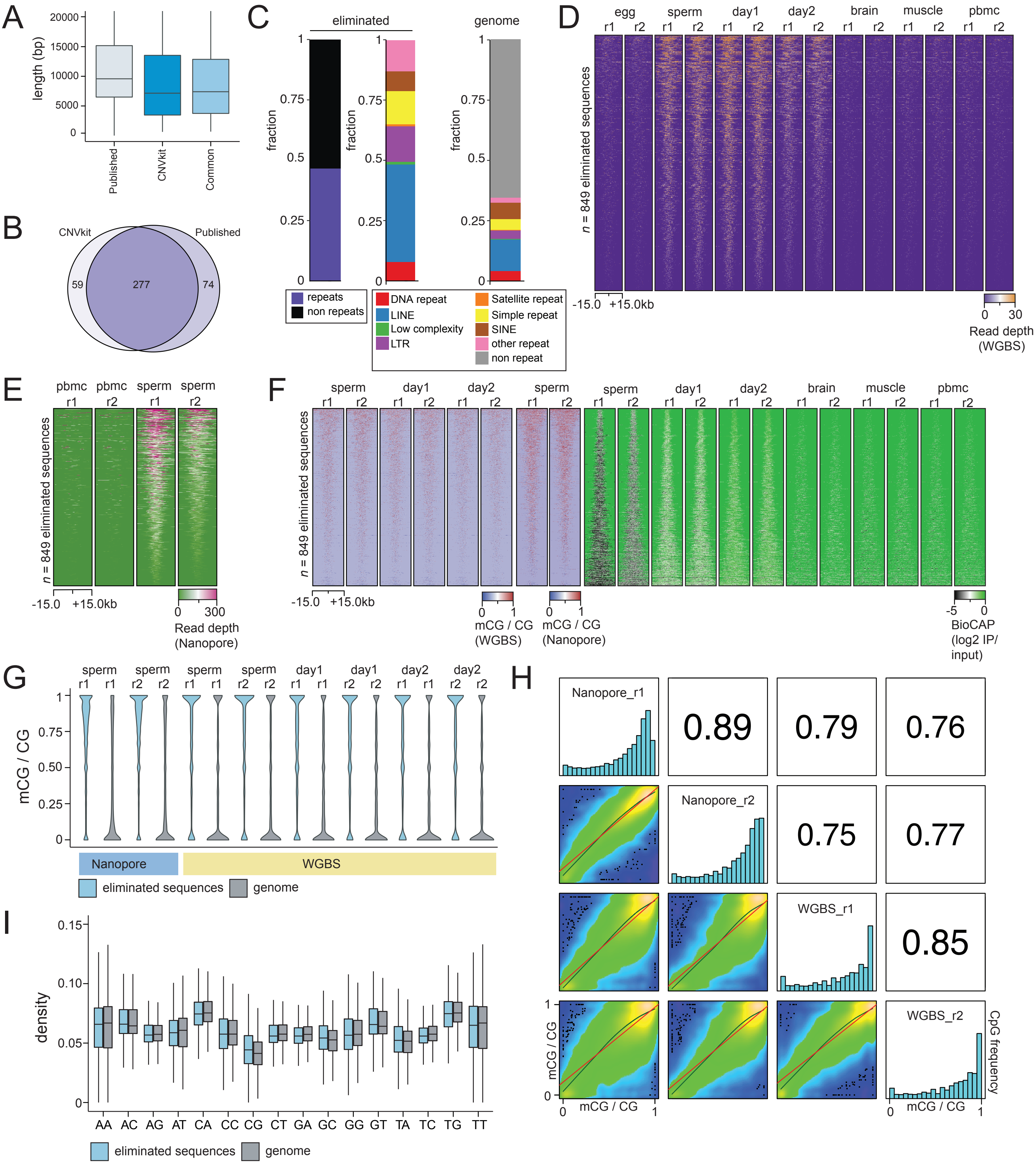


**Supplementary Figure S5. Sequence and epigenetic features of eliminated regions. A)** Distribution of lengths of previously published eliminated DNA sequences, eliminated sequences detected using CNVkit, and eliminated sequences common to both methods. The boxes show the interquartile range (IQR) around the median. The upper and lower whiskers extend from the hinge to the largest and smallest value, respectively, no further than 1.5 IQR. **B)** Number of genes in previously published eliminated DNA sequences, eliminated sequences detected using CNVkit, and eliminated sequences common to both methods. **C)** Repeat content of eliminated DNA sequences (left), repeat composition as per Repeatmasker track of eliminated DNA sequences (middle), and genomic percentage and composition of repetitive DNA sequences (Repeatmasker) in the petMar3 reference genome. **D)** Per-nucleotide read depth at eliminated sequences in bisulfite sequencing data. r1 = replicate 1, r2 = replicate 2. **E)** Per-nucleotide read depth (in biological replicate) at eliminated sequences calculated from Nanopore sequencing data. **F)** mCG and BioCAP signal (in biological replicate) at eliminated DNA sequences. **G)** mCG levels (in biological replicate) at eliminated and control sequences from Nanopore and WGBS data. **H)** Scatterplots depicting pairwise comparisons of mCG levels for Nanopore and WGBS sperm datasets. r1 = replicate 1, r2 = replicate 2. Numbers in upper right corner denote pairwise Pearson's correlation scores. Histograms on diagonal show distribution of mCG for each library. Values include CpG sites covered by all four samples, at least 5X ($n$ = 50693 CpG sites). **I)** Dinucleotide frequencies at eliminated sequences and genome-wide. The boxes show the interquartile range (IQR) around the median. The upper and lower whiskers extend from the hinge to the largest and smallest value, respectively, no further than 1.5 IQR.

| Sample | No. read pairs | No. unique deduplicated reads | Mean read depth | Bisulfite conversion rate (%) |
|---|---|---|---|---|
| Egg rep 1 | 206720490 | 158968841 | 12.85 | 98.88 |
| Egg rep 2 | 202616723 | 136606435 | 10.90 | 99.02 |
| Sperm rep 1 | 96604970 | 72771652 | 6.16 | 99.51 |
| Sperm rep 2 | 144552493 | 106935583 | 9.01 | 99.49 |
| Day 1 rep 1 | 274111966 | 155015668 | 12.33 | 99.43 |
| Day 1 rep 2 | 210912615 | 121796638 | 9.71 | 99.41 |
| Day 2 rep 1 | 225263738 | 84259234 | 7.06 | 99.76 |
| Day 2 rep 2 | 183973096 | 77455453 | 6.51 | 99.77 |
| Brain rep 1 | 86428386 | 71543799 | 6.01 | 99.39 |
| Brain rep 2 | 96879507 | 74772228 | 6.19 | 99.47 |
| Muscle rep 1 | 127337537 | 99090364 | 8.22 | 99.43 |
| Muscle rep 2 | 136701430 | 111563835 | 9.39 | 99.48 |
| PBMC rep 1 | 125107878 | 89290531 | 7.45 | 99.50 |
| PBMC rep 2 | 74522090 | 57816526 | 4.85 | 99.52 |

**Supplementary Table S1:** Read mapping information for WGBS datasets

| Sample | Experiment | No. read pairs | % unique | % multimapped | % duplicates | No. peaks |
|---|---|---|---|---|---|---|
| Sperm | Input | 68,850,331 | 34.87 | 48.05 | 9.84 | |
| | BioCAP rep 1 | 80,561,546 | 49.81 | 18.5 | 23.74 | 124,181 |
| | BioCAP rep 2 | 104,484,840 | 48.97 | 19.09 | 24.13 | 141,295 |
| Day 1 | Input | 116,902,415 | 29.2 | 39.62 | 16.18 | |
| | BioCAP rep 1 | 275,573,433 | 4.19 | 1.58 | 57.5 | 119,911 |
| | BioCAP rep 2 | 129,269,130 | 5.7 | 2.07 | 40.38 | 117,229 |
| Day 2 | Input | 204,976,559 | 18.6 | 23.11 | 16.59 | |
| | BioCAP rep 1 | 141,707,552 | 3.64 | 1.42 | 40.32 | 89,979 |
| | BioCAP rep 2 | 206,566,319 | 3.39 | 1.31 | 48.56 | 90,027 |
| Brain | Input | 81,522,370 | 40.64 | 40.62 | 13.95 | |
| | BioCAP rep 1 | 95,482,689 | 42.24 | 10.55 | 27.15 | 130,672 |
| | BioCAP rep 2 | 307,871,236 | 36.24 | 9.82 | 47.01 | 141,921 |
| Muscle | Input | 33,711,758 | 39.33 | 41.75 | 10.37 | |
| | BioCAP rep 1 | 114,586,936 | 61.16 | 15.52 | 18.04 | 140,638 |
| | BioCAP rep 2 | 285,346,303 | 55.11 | 16.04 | 21.38 | 141,334 |
| PBMC | BioCAP rep 1 | 99,098,603 | 55 | 15.98 | 15.15 | 115,448 |
| | BioCAP rep 2 | 94,230,317 | 55.38 | 16.4 | 16.56 | 117,044 |

**Supplementary Table S2: Read mapping information for bioCAP datasets**

| Run | Sample | Total output (Gb) | Passed output (Gb) | No. of reads (millions) | N50 (kb) |
|---|---|---|---|---|---|
| PBXP218432 | Sperm rep 1 | 26.32 | 21.67 | 8.31 | 6.19 |
| PBXP227438 | PBMC rep 2 | 28.02 | 25.74 | 3.97 | 18.61 |
| PBXP242452 | Sperm rep 1 | 25.41 | 23.41 | 7.32 | 6.94 |
| PBXP243452 | PBMC rep 2 | 22.94 | 19.89 | 2.62 | 23.77 |
| PBXP261461 | Sperm rep 2 | 26.94 | 19.6 | 6.22 | 7.96 |
| PBXP262461 | PBMC rep 1 | 28.37 | 23.25 | 3.51 | 20.46 |
| PBXP272467 | Sperm rep 2 | 15.3 | 6.95 | 2.65 | 9 |
| PBXP273467 | PBMC rep 1 | 27.29 | 20.3 | 3.39 | 22.55 |
| PBXP284481 | Sperm rep 2 | 23.85 | 15.32 | 5.16 | 8.81 |

**Supplementary Table S3: Read information for Nanopore datasets**
