# Supplementary material for: Extensive DNA methylome rearrangement during early lamprey embryogenesis
Source: Nat Commun. 2024 Mar 4;15:1977. doi: 10.1038/s41467-024-46085-2 (PMC10912607; doi:10.1038/s41467-024-46085-2)
Supplement: Supplementary file 3 — Description of Additional Supplementary Files [file 41467_2024_46085_MOESM3_ESM.pdf]

Description of Additional Supplementary Files

File Name: Supplementary Data 1
Description: Egg PMD coordinates (petMar3).

File Name: Supplementary Data 2
Description: Sperm PMD coordinates (petMar3).

File Name: Supplementary Data 3
Description: Day1 PMD coordinates (petMar3).

File Name: Supplementary Data 4
Description: Day2 PMD coordinates (petMar3).

File Name: Supplementary Data 5
Description: Brain PMD coordinates (petMar3).

File Name: Supplementary Data 6
Description: Muscle PMD coordinates (petMar3).

File Name: Supplementary Data 7
Description: PBMC PMD coordinates (petMar3).

File Name: Supplementary Data 8
Description: Egg non-PMD coordinates (petMar3).

File Name: Supplementary Data 9
Description: Sperm non-PMD coordinates (petMar3).

File Name: Supplementary Data 10
Description: Day1 non-PMD coordinates (petMar3).

File Name: Supplementary Data 11
Description: Day2 non-PMD coordinates (petMar3).

File Name: Supplementary Data 12
Description: Brain non-PMD coordinates (petMar3).

File Name: Supplementary Data 13
Description: Muscle non-PMD coordinates (petMar3).

File Name: Supplementary Data 14
Description: PBMC non-PMD coordinates (petMar3).

File Name: Supplementary Data 15
Description: Core NMI coordinates (petMar3).

File Name: Supplementary Data 16
Description: All DMR coordinates (petMar3).

File Name: Supplementary Data 17
Description: Eliminated sequences (petMar3).

File Name: Supplementary Data 18
Description: Genes in eliminated sequences (petMar3).
