# Supplementary material for: Extensive DNA methylome rearrangement during early lamprey embryogenesis
Source: Nat Commun. 2024 Mar 4;15:1977. doi: 10.1038/s41467-024-46085-2 (PMC10912607; doi:10.1038/s41467-024-46085-2)
Supplement: Supplementary file 5 — Reporting Summary [file 41467_2024_46085_MOESM5_ESM.pdf]

nature portfolio

Corresponding author(s): Ozren Bogdanovic

Last updated by author(s): 30 January 2024

Reporting Summary

Nature Portfolio wishes to improve the reproducibility of the work that we publish. This form provides structure for consistency and transparency in reporting. For further information on Nature Portfolio policies, see our Editorial Policies and the Editorial Policy Checklist.

Please do not complete any field with "not applicable" or n/a. Refer to the help text for what text to use if an item is not relevant to your study.
For final submission: please carefully check your responses for accuracy; you will not be able to make changes later.

Statistics

For all statistical analyses, confirm that the following items are present in the figure legend, table legend, main text, or Methods section.

n/a	Confirmed	
☐	☒	The exact sample size (*n*) for each experimental group/condition, given as a discrete number and unit of measurement
☐	☒	A statement on whether measurements were taken from distinct samples or whether the same sample was measured repeatedly
☐	☒	The statistical test(s) used AND whether they are one- or two-sided<br>*Only common tests should be described solely by name; describe more complex techniques in the Methods section.*
☐	☒	A description of all covariates tested
☐	☒	A description of any assumptions or corrections, such as tests of normality and adjustment for multiple comparisons
☐	☒	A full description of the statistical parameters including central tendency (e.g. means) or other basic estimates (e.g. regression coefficient) AND variation (e.g. standard deviation) or associated estimates of uncertainty (e.g. confidence intervals)
☐	☒	For null hypothesis testing, the test statistic (e.g. *F*, *t*, *r*) with confidence intervals, effect sizes, degrees of freedom and *P* value noted<br>*Give P values as exact values whenever suitable.*
☒	☐	For Bayesian analysis, information on the choice of priors and Markov chain Monte Carlo settings
☒	☐	For hierarchical and complex designs, identification of the appropriate level for tests and full reporting of outcomes
☐	☒	Estimates of effect sizes (e.g. Cohen's *d*, Pearson's *r*), indicating how they were calculated

Our web collection on statistics for biologists contains articles on many of the points above.

Software and code

Policy information about availability of computer code

Data collection	No software was used for data collection.
Data analysis	For WGBS mapping, data was trimmed using fastp v0.12.5 and mapped using WALT v1.0. Duplicates were removed using Picard tools v2.3.0 and 5mC calling was performed using MethylDackel v0.3.0. Bigwigs were generated using kentUtils v302.1. PMDs, UMRs and LMRs were identified using MethylSeekR v1.30.0. Genome intersections were performed using bedtools v2.29.2 or bedops v2.4.2. For BioCAP and ATAC-seq mapping, reads were trimmed using trimmomatic v0.36 and aligned using bowtie2 v2.1.0. Duplicates were removed using Picard tools v2.3.0 and peaks were called using macs2 v2.1.2. ATAC-seq reads were additionally filtered using samtools v1.11. Bigwigs were generated using deepTools v3.5.0 bamCoverage and bamCompare functions. Differentially enriched NMIs were identified using DiffBind 3.0.15. Sequence motif enrichment was performed using HOMER v4.11. DMR calling was performed using DSS. RNA-seq reads were trimmed using trimmomatic v0.36 and mapped with kallisto. Whole genome read mapping was performed using bowtie2 v2.1.0 and eliminated sequences were identified using CNVkit v0.9.10. Raw ONT sequencing data was converted to BLOW5 format using slow5tools v0.3.0 then base-called using Guppy v5.0.13 (high-accuracy model). Nanopore reads were aligned using minimap2 v2.22 and 5mC profiling was performed using f5c v0.7. CpG methylation frequencies were determined using the meth-freq tool in f5c. Read depth was identified using samtools v1.11. All reads were aligned to the petMar3 reference genome.

For manuscripts utilizing custom algorithms or software that are central to the research but not yet described in published literature, software must be made available to editors and reviewers. We strongly encourage code deposition in a community repository (e.g. GitHub). See the Nature Portfolio guidelines for submitting code & software for further information.

Data

Policy information about availability of data

All manuscripts must include a data availability statement. This statement should provide the following information, where applicable:

- Accession codes, unique identifiers, or web links for publicly available datasets
- A description of any restrictions on data availability
- For clinical datasets or third party data, please ensure that the statement adheres to our policy

Raw and processed BioCAP-seq and WGBS data generated for this study are available from NCBI Gene Expression Omnibus under accession code GSE220553 (https://www.ncbi.nlm.nih.gov/geo/query/acc.cgi?acc=GSE220553). Nanopore sequencing data generated for this study is available from NCBI under accession code PRJNA783432 (https://www.ncbi.nlm.nih.gov/bioproject/PRJNA783432). ATAC-seq data used in this study is available from NCBI Gene Expression Omnibus under accession code GSE112072 (https://www.ncbi.nlm.nih.gov/geo/query/acc.cgi?acc=GSE112072). Whole-genome sequencing data used in this study is available from NCBI Sequence Read Archive (https://www.ncbi.nlm.nih.gov/sra/) under accession codes SRR5535434 and SRR5535435. RNA-seq data used in this study is available from NCBI under accession code PRJNA50489 (https://www.ncbi.nlm.nih.gov/bioproject/50489). All sequencing data was aligned to the petMar3 reference germline genome available from NCBI under GenBank accession code GCA_002833325.1 (https://www.ncbi.nlm.nih.gov/datasets/genome/GCA_002833325.1). The Source Data underlying figures are provided as Source Data files.

Research involving human participants, their data, or biological material: N/A

Policy information about studies with human participants or human data. See also policy information about sex, gender (identity/presentation), and sexual orientation and race, ethnicity and racism.

Reporting on sex and gender	N/A
Reporting on race, ethnicity, or other socially relevant groupings	N/A
Population characteristics	N/A
Recruitment	N/A
Ethics oversight	N/A

Note that full information on the approval of the study protocol must also be provided in the manuscript.

Field-specific reporting

Please select the one below that is the best fit for your research. If you are not sure, read the appropriate sections before making your selection.

☒ Life sciences ☐ Behavioural & social sciences ☐ Ecological, evolutionary & environmental sciences

For a reference copy of the document with all sections, see nature.com/documents/nr-reporting-summary-flat.pdf

Life sciences study design

All studies must disclose on these points even when the disclosure is negative.

Sample size	All experiments were performed in biological replicate (2X) to ensure reproducibility. Each library was sequenced with sufficient depth for accurate quantification of the epigenome. No further sample size calculations were relevant for this study.
Data exclusions	No data was excluded.
Replication	All sequencing libraries were performed in biological replicate (2X). All attempts at replication were successful.
Randomization	No treatment was used in this study so randomization is not applicable.
Blinding	No treatment was used in this study so blinding is not applicable.

Behavioural & social sciences study design: N/A

All studies must disclose on these points even when the disclosure is negative.

Study description	
Research sample	
Sampling strategy	
Data collection	
Timing	
Data exclusions	
Non-participation	
Randomization	

Ecological, evolutionary & environmental sciences study design: N/A

All studies must disclose on these points even when the disclosure is negative.

Study description	
Research sample	
Sampling strategy	
Data collection	
Timing and spatial scale	
Data exclusions	
Reproducibility	
Randomization	
Blinding	

Did the study involve field work? ☐ Yes ☐ No

Field work, collection and transport: N/A

Field conditions	
Location	
Access & import/export	
Disturbance	

Reporting for specific materials, systems and methods

We require information from authors about some types of materials, experimental systems and methods used in many studies. Here, indicate whether each material, system or method listed is relevant to your study. If you are not sure if a list item applies to your research, read the appropriate section before selecting a response.

Materials & experimental systems

n/a	Involved in the study	
☒	☐	Antibodies
☒	☐	Eukaryotic cell lines
☒	☐	Palaeontology and archaeology
☐	☒	Animals and other organisms
☒	☐	Clinical data
☒	☐	Dual use research of concern
☒	☐	Plants

Methods

n/a	Involved in the study	
☒	☐	ChIP-seq
☒	☐	Flow cytometry
☒	☐	MRI-based neuroimaging

Antibodies: N/A

Antibodies used	
Validation	

Eukaryotic cell lines; N/A

Policy information about cell lines and Sex and Gender in Research

Cell line source(s)	
Authentication	
Mycoplasma contamination	
Commonly misidentified lines (See ICLAC register)	

Palaeontology and Archaeology: N/A

Specimen provenance	
Specimen deposition	
Dating methods	

☐ Tick this box to confirm that the raw and calibrated dates are available in the paper or in Supplementary Information.

Ethics oversight	

Note that full information on the approval of the study protocol must also be provided in the manuscript.

Animals and other research organisms

Policy information about studies involving animals; ARRIVE guidelines recommended for reporting animal research, and Sex and Gender in Research

Laboratory animals	All lamprey material was collected at the Hammond Bay Biological Station (Michigan, US). To produce sexually mature males and females for embryo fertilization, sea lamprey were transferred to the Ocqueoc River, Millersburg Michigan and held in cages ($0.5m^3$) to allow natural sexual maturation in a riverine environment. Sea lamprey were checked daily for sexual maturity; sexually mature individuals were identified by applying abdominal pressure and checking milt expression or for ovulated oocyte expression. Sexually mature males and female lamprey were returned to HBBS and held until use for culturing lamprey embryos. Adult lampreys used in this study were 3-5 years old.
Wild animals	No wild animals were used in this study.
Reporting on sex	Muscle and brain replicates include a male and female fish. PBMC replicates are from two male individuals. Given the high degree of similarity between male and female samples, sex was not further considered for somatic or embryonic tissues in this study.
Field-collected samples	No field collected samples were used in this study.
Ethics oversight	All experimental procedures for culturing embryos were approved by Michigan State University Institutional Animal Care and Use Committee (AUF# 02/17-031-00).

Note that full information on the approval of the study protocol must also be provided in the manuscript.

Clinical data: N/A

Policy information about clinical studies

All manuscripts should comply with the ICMJE guidelines for publication of clinical research and a completed CONSORT checklist must be included with all submissions.

Clinical trial registration	
Study protocol	
Data collection	
Outcomes	

Dual use research of concern: N/A

Policy information about dual use research of concern

Hazards

Could the accidental, deliberate or reckless misuse of agents or technologies generated in the work, or the application of information presented in the manuscript, pose a threat to:

No | Yes
- [x] No [ ] Yes Public health
- [x] No [ ] Yes National security
- [x] No [ ] Yes Crops and/or livestock
- [x] No [ ] Yes Ecosystems
- [x] No [ ] Yes Any other significant area

Experiments of concern

Does the work involve any of these experiments of concern:

No | Yes
- [x] No [ ] Yes Demonstrate how to render a vaccine ineffective
- [x] No [ ] Yes Confer resistance to therapeutically useful antibiotics or antiviral agents
- [x] No [ ] Yes Enhance the virulence of a pathogen or render a nonpathogen virulent
- [x] No [ ] Yes Increase transmissibility of a pathogen
- [x] No [ ] Yes Alter the host range of a pathogen
- [x] No [ ] Yes Enable evasion of diagnostic/detection modalities
- [x] No [ ] Yes Enable the weaponization of a biological agent or toxin
- [x] No [ ] Yes Any other potentially harmful combination of experiments and agents

Plants: N/A

Seed stocks	
Novel plant genotypes	
Authentication	

ChIP-seq: N/A

Data deposition

- [ ] Confirm that both raw and final processed data have been deposited in a public database such as GEO.
- [ ] Confirm that you have deposited or provided access to graph files (e.g. BED files) for the called peaks.

Data access links<br>*May remain private before publication.*	
Files in database submission	
Genome browser session<br>(e.g. UCSC)	

Methodology

Replicates	
Sequencing depth	
Antibodies	
Peak calling parameters	
Data quality	
Software	

Flow Cytometry: N/A

Plots

Confirm that:

- [ ] The axis labels state the marker and fluorochrome used (e.g. CD4-FITC).
- [ ] The axis scales are clearly visible. Include numbers along axes only for bottom left plot of group (a 'group' is an analysis of identical markers).
- [ ] All plots are contour plots with outliers or pseudocolor plots.
- [ ] A numerical value for number of cells or percentage (with statistics) is provided.

Methodology

Sample preparation

Instrument

Software

Cell population abundance

Gating strategy

- [ ] Tick this box to confirm that a figure exemplifying the gating strategy is provided in the Supplementary Information.

Magnetic resonance imaging: N/A

Experimental design

Design type

Design specifications

Behavioral performance measures

Imaging type(s)

Field strength

Sequence & imaging parameters

Area of acquisition

Diffusion MRI ☐ Used ☐ Not used

Preprocessing

Preprocessing software

Normalization

Normalization template

Noise and artifact removal

Volume censoring

Statistical modeling & inference

Model type and settings

Effect(s) tested

Specify type of analysis: ☐ Whole brain ☐ ROI-based ☐ Both

Statistic type for inference

(See Eklund et al. 2016)

Correction

Models & analysis

n/a	Involved in the study
☐	☐ Functional and/or effective connectivity
☐	☐ Graph analysis
☐	☐ Multivariate modeling or predictive analysis

Functional and/or effective connectivity

Graph analysis

Multivariate modeling and predictive analysis

This checklist template is licensed under a Creative Commons Attribution 4.0 International License, which permits use, sharing, adaptation, distribution and reproduction in any medium or format, as long as you give appropriate credit to the original author(s) and the source, provide a link to the Creative Commons license, and indicate if changes were made. The images or other third party material in this article are included in the article's Creative Commons license, unless indicated otherwise in a credit line to the material. If material is not included in the article's Creative Commons license and your intended use is not permitted by statutory regulation or exceeds the permitted use, you will need to obtain permission directly from the copyright holder. To view a copy of this license, visit http://creativecommons.org/licenses/by/4.0/
